# Erythropoietic protoporphyrias: Pathogenesis, diagnosis and management

**DOI:** 10.1111/liv.16027

**Published:** 2024-07-16

**Authors:** Anna‐Elisabeth Minder, Louisa G. Kluijver, Jasmin Barman‐Aksözen, Elisabeth I. Minder, Janneke G. Langendonk

**Affiliations:** ^1^ Division of Endocrinology, Diabetology, and Porphyria Stadtspital Zürich Triemli Zurich Switzerland; ^2^ Swiss Reference Centre for Porphyrias Stadtspital Zürich Triemli Zurich Switzerland; ^3^ Department of Internal Medicine, Porphyria Center Rotterdam, Center for Lysosomal and Metabolic Disease, Erasmus MC University Medical Center Rotterdam The Netherlands; ^4^ Institute of Laboratory Medicine Stadtspital Zürich Triemli Zurich Switzerland; ^5^ University of Zurich Zurich Switzerland

**Keywords:** afamelanotide, cholestatic liver disease, CLPX‐protoporphyria, EPP, erythropoietic protoporphyria, painful skin photosensitivity, protoporphyria‐related liver damage, X‐linked protoporphyria

## Abstract

The erythropoietic protoporphyrias consist of three ultra‐rare genetic disorders of the erythroid heme biosynthesis, including erythropoietic protoporphyria (EPP1), X‐linked protoporphyria (XLEPP) and CLPX‐protoporphyria (EPP2), which all lead to the accumulation of protoporphyrin IX (PPIX) in erythrocytes. Affected patients usually present from early childhood with episodes of severe phototoxic pain in the skin exposed to visible light. The quantification of PPIX in erythrocytes with a metal‐free PPIX ≥3 times the upper limit of normal confirms the diagnosis. Protoporphyria‐related complications include liver failure, gallstones, mild anaemia and vitamin D deficiency with reduced bone mineral density. The management is focused on preventing phototoxic reactions and treating the complications. Vitamin D should be supplemented, and DEXA scans in adults should be considered. In EPP1, even in cases of biochemically determined iron deficiency, supplementation of iron may stimulate PPIX production, resulting in an increase in photosensitivity and the risk of cholestatic liver disease. However, for patients with XLEPP, iron supplementation can reduce PPIX levels, phototoxicity and liver damage. Because of its rarity, there is little data on the management of EPP‐related liver disease. As a first measure, any hepatotoxins should be eliminated. Depending on the severity of the liver disease, phlebotomies, exchange transfusions and ultimately liver transplantation with subsequent haematopoietic stem cell transplantation (HSCT) are therapeutic options, whereby multidisciplinary management including porphyria experts is mandatory. Afamelanotide, an alpha‐melanocyte‐stimulating hormone analogue, is currently the only approved specific treatment that increases pain‐free sunlight exposure and quality of life.

**Abbreviations**
ALAS1: 5‐aminolevulinic acid synthase 1
ALAS2: 5‐aminolevulinic acid synthase 2
CEP: congenital erythropoietic porphyria
EPP1: erythropoietic protoporphyria
EPP2: CLPX‐protoporphyria
FECH: ferrochelatase
HCP: hereditary coproporphyria
Ipnet: International Porphyria Network
IRP2: iron‐responsive element binding protein 2
MC1R: melanocortin receptor 1
OMIM: online Mendelian inheritance in man
PASS: post‐authorisation safety and efficacy study
PCT: porphyria cutanea tarda
PPIX: protoporphyrin IX
ROS: reactive oxygen species
VP: variegate porphyria
XLEPP: X‐linked protoporphyria
ZnPP: zinc protoporphyrin IX

**Key points**

1. The protoporphyrias consisting of erythropoietic protoporphyria, CLPX‐protoporphyria and X‐linked protoporphyria are caused by mutations in the ferrochelatase, CLPX and 5‐aminolevulinic acid synthase 2 genes, respectively.
2. The main symptom is severe phototoxic pain due to the accumulation of excess protoporphyrin IX (PPIX) in skin exposed to visible light.
3. The current management consists of light protection and the alpha‐melanocyte‐stimulating‐hormone analogue afamelanotide.
4. Due to the biliary elimination of excess PPIX, the most severe complication is cholestatic liver disease, which may progress into terminal failure.
5. Treatment strategies for cholestatic liver disease are based on the elimination of any hepatotoxins. The evidence for further options in advanced liver failure is based on case reports and is discussed in this review.

## 1 INTRODUCTION

The protoporphyrias are a group of three ultra‐rare genetic disorders of the erythroid heme biosynthesis (prevalence between 1:75000 and 1:100000), all leading to the accumulation of protoporphyrin IX (PPIX) in erythrocytes. The group of protoporphyrias includes erythropoietic protoporphyria (OMIM: erythropoietic protoporphyria [EPP1], #177000), X‐linked protoporphyria (OMIM: XLEPP, #300752) and CLPX‐protoporphyria (OMIM: EPP2, #618015), which are caused by mutations in three different genes related to this biosynthesis pathway. Patients generally present themselves from early childhood on with episodes of severe phototoxic pain in skin exposed to visible light, especially the hands, forearms, feet and face. The clinical picture of protoporphyria was first described in 1961 by Magnus et al.^1^ whereas the complex mode of inheritance, EPP1, the most prevalent form of protoporphyria, was only described in 1984,^2^ and the unique genetic background was resolved in 2002,^3^ more than 40 years after its first description. Moreover, recent research led to the identification of the distinct diseases XLEPP and EPP2. Many features of the three protoporphyrias are similar; however, some treatment aspects differ according to the specific subtype.

In this review, we focus on the clinical picture, pathogenesis, inheritance pattern and diagnostic procedures of EPP1 and XLPP. Furthermore, we are outlining the treatment and management of the disease and its complications according to current knowledge and our clinical expertise.

The authors used their expertise to decide on the discussed topics, for which the current scientific standard was collected using local article databases and then ascertained by extensive PubMed and Google scholar searches as per discussions of the author experts.

## 2 PATHOGENESIS

### 2.1 Heme biosynthesis

Heme is an essential cofactor of many proteins in the organism, such as haemoglobin and other haemoproteins, including myoglobin, cytochromes, catalases and heme peroxidases. Therefore, heme biosynthesis occurs in almost all living cells, with the vast majority, approximately 80% of total body heme, synthesized in the erythroid cells of the bone marrow in order to produce haemoglobin.

**FIGURE 1:**
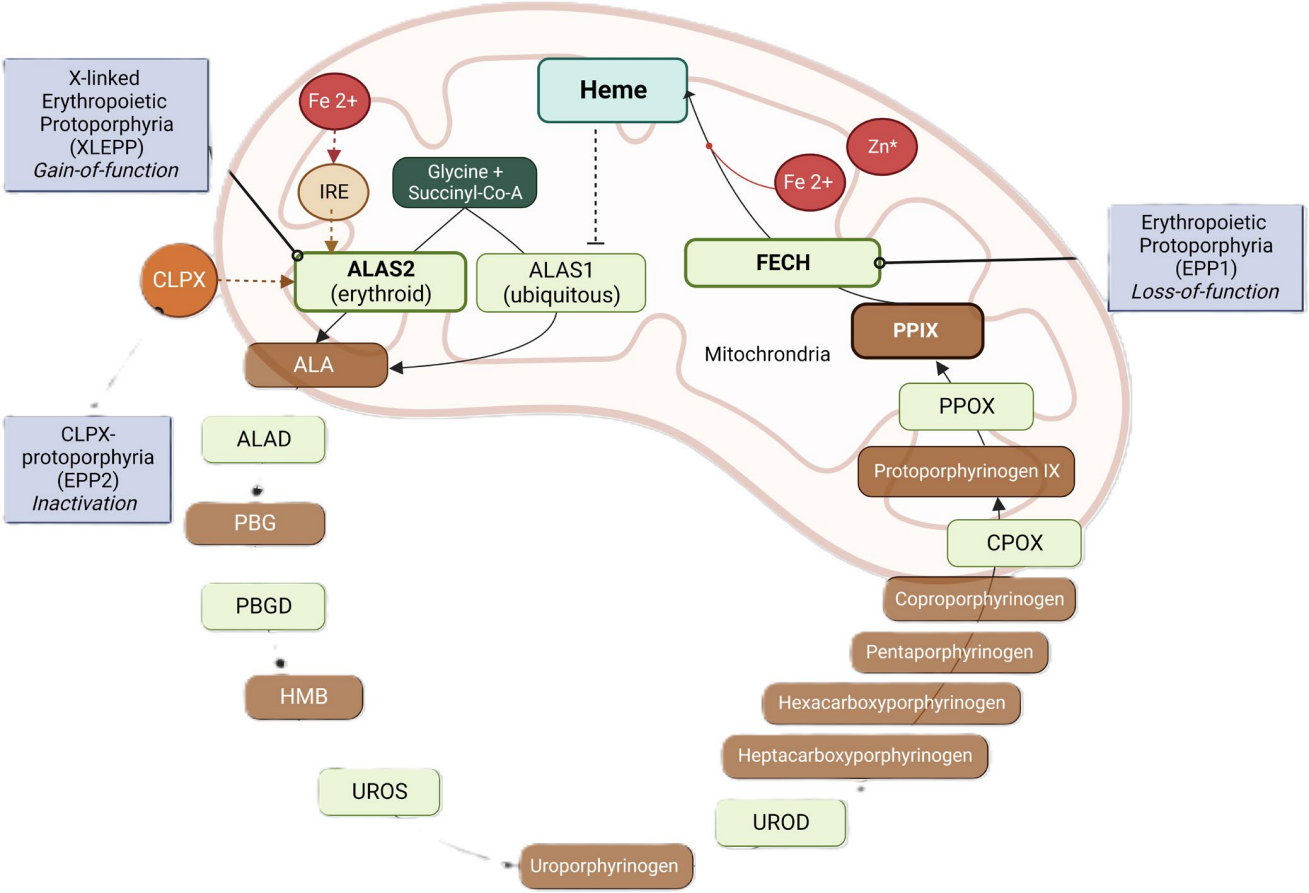
Heme biosynthesis pathway and the corresponding defects in enzymes leading to protoporphyrias. The last enzyme of the heme biosynthesis, FECH, inserts ferrous iron (Fe2+) into PPIX‐forming heme. In EPP1, loss‐of‐function mutations in FECH lead to reduced conversion and ultimately accumulation of PPIX, causing the protoporphyria phenotype. The first enzyme within the pathway, ALAS, has two isoforms: ALAS2 in the erythroid cells, positively regulated by iron, and ALAS1 in all other tissues, negatively regulated by heme. Increased ALAS2 enzyme activity can be caused by either gain‐of‐function mutation in the ALAS2 gene (XLEPP) or partial inactivation in the CLPX gene (EPP2). Both lead to activation of the heme biosynthesis according to iron availability with FECH being a rate‐limiting step, ultimately leading to the accumulation of PPIX. Iron supplementation (Fe2+) can also stimulate ALAS2 translation, increasing its activation, leading to increased PPIX in EPP1. In XLEPP, iron supplementation has the opposite effect leading to a decrease in PPIX. * In situations with low ferrous iron (Fe2+) availability, often seen in XLEPP, FECH catalyses the insertion of zinc (Zn^2+^) into PPIX, creating Zinc‐protoporphyrin IX (ZnPP). ALA, 5‐aminolevulinic acid; ALAD, ALA‐dehydratase; ALAS1, ALA‐synthase 1; ALAS2, ALA‐synthase 2; CLPX, mitochondrial AAA+ unfoldase CLPX; CPOX, copro‐oxidase; EPP1, erythropoietic protoporphyria; FECH, ferrochelatase; HMB, hydroxymethylbiliane; PBG, porphobilinogen; PBGD, PBG‐deaminase; PPOX, proto‐oxidase; UROD, Uro‐decarboxylase; UROS, Uro‐III‐synthase.

Heme biosynthesis consists of a tightly regulated, eight‐step enzymatic pathway (Figure 1). In the protoporphyrias, the enzymatic defects manifest in the erythropoiesis and affect either the initial step in the case of XLEPP and EPP2, or the final step in the case of EPP1.

The first step of heme biosynthesis involves converting glycine and succinyl‐CoA to 5‐aminolevulinic acid (ALA), catalysed by either the ubiquitous 5‐aminolevulinic acid synthase 1 (ALAS1) enzyme or the erythroid‐specific 5‐aminolevulinic acid synthase 2 (ALAS2) enzyme. The latter is encoded on the X‐chromosome by a gene different from the ubiquitous first enzyme of heme biosynthesis, ALAS1.^4^, ^5^ In the liver, the rate of heme synthesis is regulated by ALAS1 via feedback mechanisms according to the intracellular heme requirements, whereas in erythroid cells, the rate is determined by ALAS2 and the availability of iron.^4^, ^6^ One of the regulators of the ALAS2 enzyme is the mitochondrial AAA+ unfoldase alternative ATPase subunit CLPX (CLPX). The final step of erythroid heme biosynthesis is catalysed by the ferrochelatase (FECH) enzyme and involves the insertion of ferrous iron into the PPIX ring, leading to the formation of heme.^4^

### 2.2 Light‐induced skin damage caused by PPIX

As a precursor of heme, PPIX is physiologically present in all living cells. However, in any of the protoporphyrias, pathological variants in the ALAS2, CLPX or FECH genes lead to the accumulation of excess PPIX in the maturating erythroblasts in the bone marrow. After the maturation of the reticulocytes and their release into the blood stream, the excess PPIX diffuses from the erythrocytes into the plasma, where it is bound to albumin.^7^ Irradiation by light may further promote the release of PPIX from the erythrocytes.^8^

**FIGURE 2:**
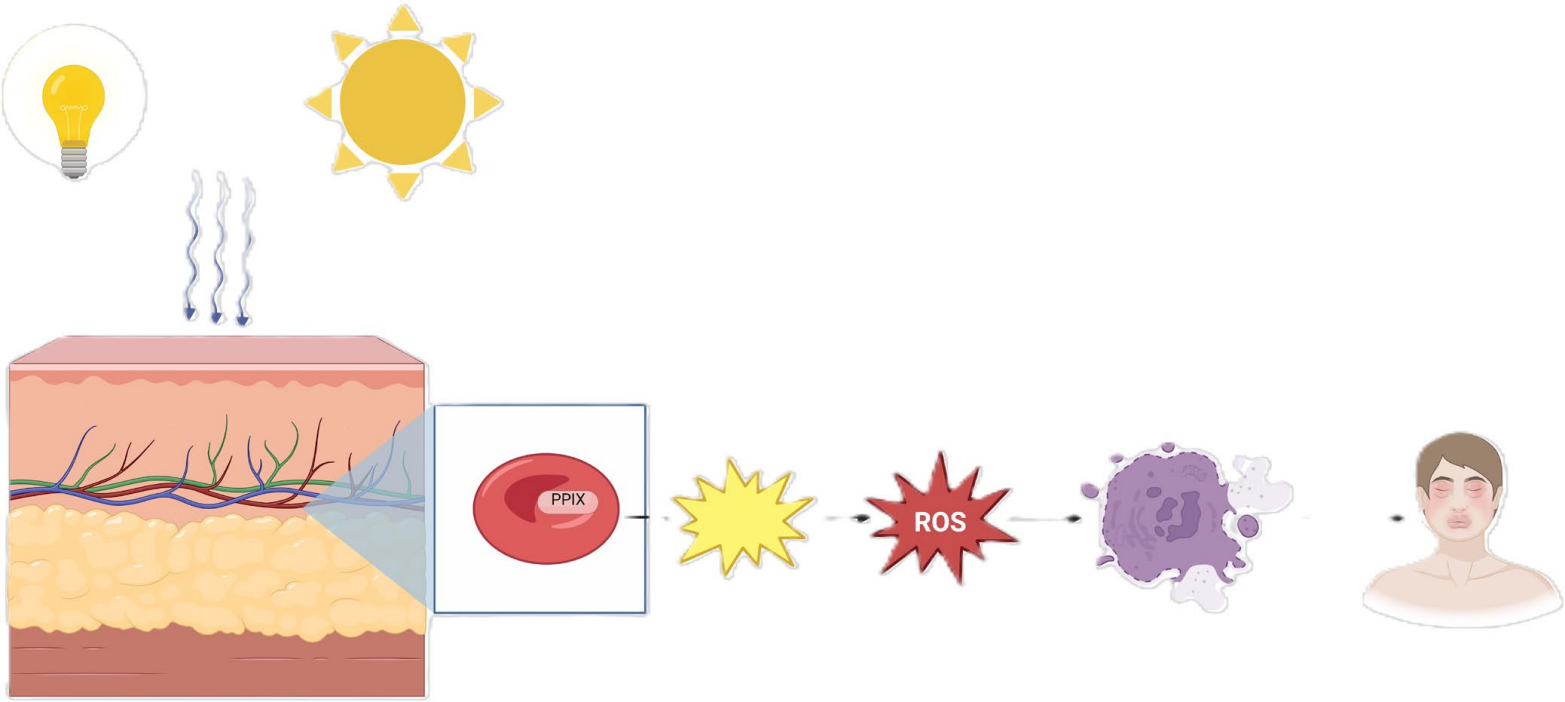
Photoactivation of protoporphyrin IX in protoporphyrias. This figure illustrates the absorption of visible light by PPIX, leading to photoactivation. Visible light from both indoor light sources and sun exposure can penetrate the skin, reaching the superficial capillaries containing PPIX‐filled erythrocytes. The light‐activated PPIX transfers its energy to molecular oxygen, generating reactive oxygen species (ROS). These toxic oxygen products cause damages to cellular structures, resulting in pain, oedema and erythema of the (sun)light‐exposed skin.

Isolated PPIX absorbs the radiation energy in the visible light spectrum, predominantly blue light at 409 nm, which alters the energy state of PPIX.^9^ Additional absorbance bands are present between 580 and 650 nm, and in the infrared region.^10^ The light‐activated PPIX molecule transfers its energy to molecular oxygen, generating reactive oxygen species (ROS). These toxic oxygen products cause damage to the surrounding cellular structures (Figure 2). However, the experimentally determined absorbance spectrum does not necessarily represent the in vivo absorption in the skin or the action spectrum of singlet oxygen formation and, therefore, might not accurately represent the wavelength where the most damage occurs.^11^, ^12^ Histologically, the subpapillary vascular endothelial cells and the basal membranes are most harmed, resulting in an activation of the complement system,^13^ local inflammation and necrosis, which is considered to explain the painful phototoxic burns after exposure to visible light.^14^

### 2.3 Genetic inheritance

**FIGURE 3:**
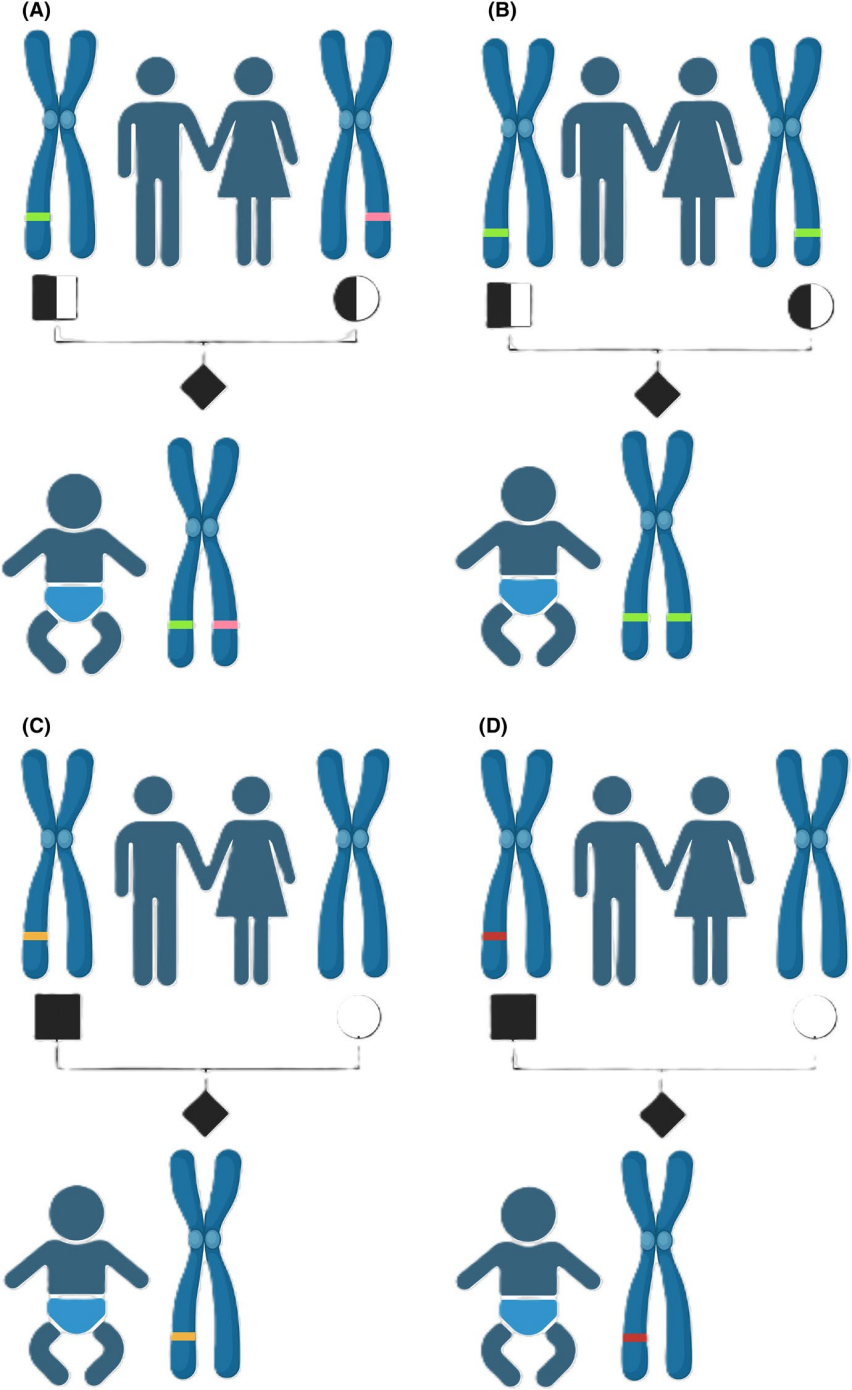
Inheritance pattern of the protoporphyrias. The four different inheritance patterns of the protoporphyrias are shown above. (A) EPP1: Illustrates the most prevalent form of inheritance of protoporphyrias, consisting of a FECH loss‐of‐function mutation in trans to a common low expression allele. This is considered a compound heterozygous, autosomal recessive form of inheritance. This leads to a loss‐of‐function of the enzyme, with a residual FECH activity of around 30%. *Homozygous inheritance of the common low expression allele does not lead to EPP1. (B) ‘Homozygous or compound heterozygous’ EPP1: This term is used for rare cases of homozygous or compound heterozygous inheritance of FECH mutations on both alleles. This leads to a severely reduced FECH enzyme activity, depending on the mutations. (C) XLEPP: Illustrates the X‐linked dominant inheritance of the ALAS2 gain‐of‐function mutation, seen in both male and female cases. This leads to increased activity of the first enzyme of heme synthesis, with unaffected ‘normal’ FECH activity. FECH is a rate‐limiting step, resulting in PPIX accumulation. (D) EPP2: Illustrates the autosomal dominant inheritance of CLPX, a stabilizing protein of ALAS2. Inactivation of this protein leads to increased ALAS2 enzyme activity, similar to XLEPP. Nomenclature is based on the OMIM database categories, which is an online catalogue of human genes and genetic disorders, updated version 26 January 2024. ALAS1, 5‐aminolevulinic acid synthase 1; ALAS2, 5‐aminolevulinic acid synthase 2; EPP1, erythropoietic protoporphyria.

Over 90% of the patients with protoporphyria suffer from EPP1. The prevailing cause of EPP1 is the presence of a loss‐of‐function mutation in one *FECH* allele combined with a heteroallelic low‐expression allele *in trans* (Figure 3A). Inheriting both the mutation and the low‐expression allele lowers the FECH activity to around 30% of normal.^15^ The low‐expression allele carries the common splice‐altering variant *FECH* c.315‐48T>C (former annotation: IVS3‐48T/C).^16^ Approximately 5%–10% of the European Caucasians population is a carrier for the low expression allele, which is even more prevalent in Japan (43%), and southeast Asia (31%), and practically absent in populations of sub‐Saharan origin.^3^, ^16^, ^17^, ^18^, ^19^ Of note, homozygosity of this low‐expression allele does not result in the phenotype of protoporphyria.

Rarely, individuals with protoporphyria carry homozygous or compound heterozygous missense mutations in the *FECH* gene (Figure 3B) with an autosomal recessive inheritance pattern. In the described cases of recessive EPP1, this resulted in a more pronounced decrease in FECH activity compared to EPP1 with the low expression allele.^15^ The blood PPIX concentrations are variable between the described patients, depending on the specific mutations, the residual FECH activity and the iron status. In addition to the typical clinical protoporphyria symptoms, palmar keratoderma is observed in homozygous EPP1.^20^

About 4%–10% of protoporphyria patients suffer from XLEPP, which shows an X‐linked dominant inheritance.^21^, ^22^ XLEPP is caused by a gain‐of‐function mutation of the ALAS2 gene (Figure 3C). Thus far, only gain‐of‐function mutations in exon 11 have been identified to cause XLEPP. As a gain‐of‐function mutation, XLEPP is present in both sexes, although the penetrance can be altered by X chromosome inactivation in females.^21^, ^23^ The gain‐of‐function mutation results in increased ALAS2 enzyme activity and high levels of PPIX in erythroid cells.^21^, ^22^, ^24^ Within the erythrocytes, the total PPIX is high, including a large proportion of zinc PPIX (ZnPP)^15^, ^24^ (Table 1), whereby this proportion depends on the iron status, as explained later.

**TABLE 1:** The differences between protoporphyrias.

	EPP1	XLEPP	EPP2
Proportion ZnPP/total PPIX	Low <15%	High 19%–65%	High 29%–67%
Effect of iron supplementation on PPIX level	Increase	Decrease	Unknown^a^
Inheritance	Autosomal recessive	X‐Linked dominant	Autosomal dominant
Enzyme activity	Decreased FECH	Increased ALAS2	CLPX inactivation → increased ALAS2

Abbreviations: ALAS2, 5‐aminolevulinic acid synthase 2; EPP1, erythropoietic protoporphyria; FECH, ferrochelatase; ZnPP, zinc protoporphyrin.

^a^ Decrease expected.

In addition, in 2017, a pathogenic variant in the *CLPX* gene was described as causing protoporphyria with an autosomal‐dominant inheritance in a family (Figure 3D).^25^ The *CLPX* gene codes for a mitochondrial AAA+ unfoldase, or the alternative ATPase subunit CLPX. A normally functioning CLPX degrades ALAS1 and ALAS2. Partial inactivation of the CLPX gene by a mutation has been shown to result in an increase in posttranslational stability of the ALAS2 protein and therefore increased ALAS2 activity, resulting in EPP2, with similar clinical and biochemical characteristics as XLEPP.^26^

Table 1 summarizes the inheritance and the differences in biochemistry among the different protoporphyrias.

Lastly, an extremely rare, acquired adult onset of protoporphyria in the setting of a myelodysplastic or myeloproliferative syndrome has been reported.^27^ In these patients, clones of erythroid progenitors carrying abnormalities or de novo mutations in chromosome 18q, the locus of FECH, are present. Following clone expansion due to tumour growth, these tumour cells can produce significant amounts of PPIX, causing the protoporphyria phenotype. As this is a paraneoplastic phenomenon, the successful treatment of the underlying condition may cure the protoporphyria.^27^

The onset of symptoms in adulthood is usually related to this acquired paraneoplastic protoporphyria; however, genetically‐based protoporphyria with initial symptoms emerging in adulthood has also been reported.^28^

## 3 CLINICAL PRESENTATION

Pain confined to the light‐exposed skin during and after light irradiation is characteristic of protoporphyria. The majority of patients recall that they had their first symptoms before the age of 5 years.^22^, ^29^, ^30^, ^31^ There is, however, often a significant diagnostic delay,^29^, ^30^, ^31^ with some patients only being diagnosed in late adulthood. The main hurdle for an early diagnosis is the lack of objective and visible skin alterations during many of the painful phototoxic reactions. Since most children may not be able to articulate their symptoms, parents often notice signs like increased irritability, crying or allergy‐like manifestations, including oedema and swelling. These symptoms manifest on light‐exposed skin; predominantly on the face, dorsal hands, forearms and dorsal feet.

Early symptoms often manifest within a few minutes of light exposure as tingling, itching or burning, which intensify with prolonged light exposure.^32^ For some patients, these symptoms are warning signals to exit the sun and prevent phototoxic reactions,^32^ while for others, they indicate the onset of the phototoxic reaction. The phototoxic reaction is characterized by severe pain,^33^, ^34^ although the skin typically exhibits no visible alterations within the first few minutes to hours. Instead, these alterations become apparent hours to days after the onset of pain, if at all. Cutaneous manifestations at a later stage may include erythema, oedema, wounds, crusts, petechiae and, in extreme cases, necrosis.^35^ Though they may occur, blisters or vesicles are rare,^35^ and more commonly associated with other forms of cutaneous porphyrias.

The onset of symptoms may vary, occurring after minutes or hours of light exposure,^33^ depending on weather conditions and on the patient's individual light tolerance.^30^ The duration of painful phototoxic reactions can span from hours to several days,^35^ depending on recent light exposures and the duration of time in the sun.^36^ Repeated light exposure can increase light sensitivity, the so‐called ‘priming phenomenon’.^36^ The primary provoking factor is visible light, and sunlight is the most potent source, but symptoms can also emerge indoors from artificial light sources,^29^ or after exposure to light through glass windows,^35^ whether at home, on the bus or train, or at school or work, posing substantial challenges for patients.

As previously mentioned, symptom onset depends on weather conditions, with some patients reporting a decrease in light tolerance and/or exacerbation of phototoxic reactions in hot, cold and windy conditions.^29^, ^31^ Some patients report reduced symptoms when they are near the equator, as recounted anecdotally in clinical practice. Besides weather conditions, several serious provoking factors for the increased severity of symptoms are known. Both iron supplementation in EPP1^37^ and cholestatic liver disease^22^, ^38^ are associated with increasing PPIX and photosensitivity. On the contrary, pregnancy is known to decrease photosensivity,^29^, ^31^ causing an unexplained decrease in PPIX.^39^

Patients consistently report a low quality of life,^30^, ^40^, ^41^, ^42^ particularly impacting social functioning but also affecting physical and mental health, vitality, bodily pain and general health.^30^ Additionally, employment rates are lower despite higher education levels when compared to the general population.^30^

### 3.1 Diagnosis

#### 3.1.1 Laboratory testing

Individuals experiencing pain in light‐exposed skin should undergo testing for protoporphyria.

In general, the biochemical analyses should be performed in a laboratory acknowledged as a specialized centre for porphyrin diagnostics, such as the laboratories certified by the International Porphyria Network (Ipnet, formerly known as European Porphyria Network; porphyrianet.org.). These laboratories can also give advice regarding the optimal laboratory testing or differential diagnosis of biochemical abnormalities. As sample materials and pre‐analytical specifications depend on the specific diagnostic methods, it is advisable to contact the respective laboratory prior to sample collection.

For the initial screening for porphyrias with cutaneous symptoms, a plasma fluorescence scan detecting increased plasma porphyrin concentrations is recommended. This approach offers the added benefit of recognizing other forms of porphyrias associated with cutaneous symptoms, that is, porphyria cutanea tarda (OMIM: PCT, #176100), porphyria variegata (OMIM: VP, #176200), hereditary coproporphyria (OMIM: HCP, #121300) and congenital erythropoietic porphyria (OMIM: CEP, #263700). Moreover, a negative test result excludes any form of porphyria as the underlying cause of concurrent skin symptoms. In the case of the protoporphyrias, PPIX present in the plasma typically causes a positive plasma‐fluorescence scan with a peak at around 633–635 nm.^43^ The diagnosis of the protoporphyrias is confirmed by the quantification of PPIX in erythrocytes, whereby metal‐free PPIX and ZnPP are determined separately. A metal‐free PPIX ≥3 times the upper limit of normal (ULN) establishes the diagnosis of protoporphyrias.

To reliably perform a plasma fluorescence scan and the PPIX measurement in erythrocytes, the vial of blood (5 mL) needs to be protected from light with an aluminium foil. Exposure of the tube to light can lead to photobleaching of PPIX, resulting in a decrease in measured PPIX levels in the sample as compared to the actual levels in the subject. This effect is related to the ability of conjugated double bonds in the porphyrin rings to absorb the energy from visible light.^44^

Other test materials used for the diagnosis of other forms of porphyrias are urine and faeces. Urinary porphyrins are not diagnostic in protoporphyrias as, due to its hydrophobicity, excess PPIX is excreted by the biliary route and faeces. Therefore, in the faeces, an increase in PPIX can be present, but the diagnosis requires confirmation by a significant increase of PPIX and ZnPP in the erythrocytes.

#### 3.1.2 Biochemical discrimination of EPP1, XLEPP and EPP2

FECH catalyses the insertion of iron into PPIX. If iron availability is low, it also catalyses the insertion of zinc into PPIX to form ZnPP (Figure 1, Table 1). Therefore, in EPP1, predominantely metal‐free PPIX accumulates. In the case of XLEPP, with increased ALAS2 activity, iron becomes the rate‐limiting substrate for the erythropoietic heme synthesis, leading to PPIX accumulation. However, as FECH activity is not affected in XLEPP, zinc can still be effectively inserted into PPIX, leading to an accumulation of both PPIX and ZnPP. Therefore, XLEPP can usually be distinguished biochemically from EPP1 by the higher proportion of ZnPP. In XLEPP, the proportion of ZnPP ranges between 19% and 65% of total PPIX, whereas ZnPP is typically <15% of total PPIX in EPP1.^21^, ^45^ In the one family with EPP2, an increase in both PPIX and ZnPP was observed (Table 1).

#### 3.1.3 Genetic testing

Routine sanger sequencing methods as well as high‐throughput sequencing have been established for the protoporphyrias. A molecular diagnosis can confirm the specific diagnosis and differentiate between the three different types of protoporphyrias. Family counselling is another purpose for genetic testing. It should be noted that due to variable reasons, such as insufficient sequence coverage, complex inheritance or the detection of variants with unknown significance, a genetic test result does not definitely diagnose or exclude any type of porphyria. For protoporphyrias, an increased blood PPIX concentration is the hallmark of a diagnosis.

#### 3.1.4 Skin biopsy

Skin biopsies are not required for the diagnosis of protoporphyrias or any other type of cutaneous porphyria. The biochemical tests described above are sufficiently sensitive and specific to diagnose this metabolic condition. If a skin biopsy is performed, the histology varies depending on the phase of the phototoxic reaction. In the acute phase, a non‐specific, intense perivascular and interstitial, largely neutrophilic dermal infiltrate, as well as visible endothelial damage to the dermal superficial vessels, is present. More chronically, there is non‐specific epidermal small vessel damage with the deposition of amorphous PAS‐positive hyaline material in the blood vessel wall of the upper and the papillary dermal vascular plexuses, which are markedly thickened. Also, there is an immunoglobulin G deposition in the immunofluorescence studies.^46^, ^47^, ^48^, ^49^

## 4 MANAGEMENT

The primary aim in the management of protoporphyrias is focused on preventing phototoxic reactions and patient support.

### 4.1 Prevention of phototoxic reactions and patient support

Without access to specific treatment options that prevent or treat phototoxic reactions, patients can only adapt their behaviour by avoiding activities outdoors in sunlight and by using light filters and photo‐protective clothing consisting of opaque clothing with long sleeves, headwear, gloves, enclosed footwear and shields, even during the winter months. Given the early onset of the protoporphyrias and the diagnostic delay, most patients are already conditioned to avoid (sun)light before their diagnosis. In contrast to UV radiation, visible light has the ability to penetrate through glass. Commercially available window shields and filters can effectively mitigate this penetration. Information on these shields can be obtained from patient organizations and porphyria expert centres. Patients increasingly report that currently used artificial light sources can cause phototoxic reactions indoors. Therefore, indoor adaptations may include the installation of tolerable light sources. While these measures can be implemented in personal environments, it remains challenging to adapt to the school and work environments of the patients. Moreover, it remains nearly impossible to implement protective measures in public areas, including public transportation. It is crucial and complex for protoporphyria patients and their families to inform and educate their social network, to raise awareness and understanding of the limitations and the necessary support.^50^ Supportive care can include a document and/or medical alert card from the porphyria centre describing the medical condition, as well as the consequences of light exposure and the need for light protection. With these documents, patients can inform not only their peers, teachers and colleagues but also their health care providers, enabling a better understanding of the required adaptations. Support should include the provision of sufficient background information and official letters to address protoporphyria‐related issues in daily life. In newly diagnosed patients, efforts should be made to educate patients on effective light protective measures and protocols for surgical light use. Lastly, they should be informed about inheritance and options for genetic counselling.

As described in the treatment section below, in some countries, adult patients can be treated with afamelanotide, the thus far only approved pharmacological treatment to prevent the phototoxic reactions in protoporphyrias.

### 4.2 Management of pain during phototoxic reactions

There are no effective analgesics to alleviate the severe pain associated with the phototoxic reactions. In one cross‐sectional questionnaire study, patients reported employing various measures to relieve pain caused by acute phototoxic reactions.^29^ For some relief, nearly all patients used wet towels, ice, cold water or cold objects. Some mentioned the use of fans, lotions, cooling balms, creams containing cortisone, alcohol and herbs. None of these measures were reported to be particularly effective. Anecdotally, patients reported heat applications (such as hot water or a sauna) to be effective in alleviating the pain after an initial exacerbation of symptoms. The effectiveness of these measures may vary for each individual and should be evaluated by the patients themselves.

The recovery from a phototoxic reaction may last several days and includes the avoidance of light and heat.

### 4.3 Perioperative measures during surgery

An old study from 2008 showed that short surgical procedures could be performed without additional action.^51^ However, in patients with medium to high PPIX or signs of liver involvement, prolonged surgeries can be challenging.^52^, ^53^ According to some case reports, surgical lamps have caused severe phototoxic burns, necrosis of the skin and internal organs, hemolysis,^54^ and even death.^55^

As a precautionary measure, physicians performing surgery or endoscopic intervention should therefore be advised to limit their exposure to light during the procedure as best as possible, and the patient should be shielded from light postoperatively to prevent painful phototoxic reactions by closing curtains as a simple measure.^56^ Filters blocking the most harmful visible light have also been used successfully in preventing phototoxic burns during and after surgery.^56^, ^57^ Also, in severe cases, erythrocyte exchange transfusions have been used preoperatively as a preventive measure.^53^, ^56^

### 4.4 Psychological health

As previously mentioned, protoporphyrias have a significant impact on the quality of life, particularly leading to isolation, social inhibition and decreased social wellbeing. This is associated with higher risks of anxiety and depression.^30^ The impact of this disease on mental health and the psychosocial impact should be taken into account, and a consultation with a psychologist experienced in protoporphyrias may be considered.

### 4.5 Counselling regarding inheritance

Depending on the type of protoporphyria, the inheritance pattern can be either autosomal recessive, X‐linked dominant, or autosomal dominant (Figure 3). A complicating factor is the variation in the prevalence of the low expression allele in the *FECH* gene in the populations, as previously discussed in the genetic inheritance section. Preconception counselling on genetic testing should be offered to both, patients and asymptomatic relatives.

### 4.6 Treatment

#### 4.6.1 Afamelanotide

There is currently only one effective and approved symptomatic treatment for the prevention of phototoxic reactions in adult patients, the alpha‐melanocyte‐stimulating hormone (α‐MSH) analogue afamelanotide (Nle4‐D‐Phe7‐α‐melanocyte‐stimulating hormone).^58^ It stimulates eumelanin production, which is photoprotective, has anti‐inflammatory properties and scavenges free radicals.^40^ Both randomized controlled trials and long‐term observational studies, have shown that it statistically significantly increases the time spend in direct sunlight without pain and also significantly improves the quality of life of patients with protoporphyrias.^40^, ^41^, ^59^ In addition to demonstrating good clinical efficacy, afamelanotide also has a favourable safety profile. The reported side‐effects are mild and include skin hyperpigmentation, diarrhoea, nausea and headaches.^40^ Its long‐term safety has been documented in the mandatory post‐authorisation safety and efficacy study (PASS), which was setup following the approval of afamelanotide in the EU in 2014 by the EMA.^41^, ^59^ After the regulatory approval in the EU, afamelanotide has also been approved for the treatment of adults in other countries, for example, the USA (2019) and Australia (2020).

#### 4.6.2 Other pharmaceutical treatment options

There is an extensive and complete review on previous drugs such as beta‐carotene and cimetidine;^60^, ^61^ however, none of these have any significant effect on protoporphyrias.^62^, ^63^ There are two new drugs on the horizon: dersimelagon, currently in phase III trials,^64^ and bitopertin, in phase II.^65^

Dersimelagon is a melanocortin receptor 1 (MC1R) agonist with a similar mode of action to afamelanotide. In contrast to afamelanotide, which has to be given as a subcutaneous implant, this drug is available in an oral form. A similar side effect profile as afamelanotide, including nausea, freckles, headaches and skin hyperpigmentation, has been reported.^64^ The first published phase II trial results demonstrated effectiveness,^64^ while the phase III trial did not meet its primary efficacy endpoint (clinical trial.gov, ID NCT04440592 and NCT05005975).

The bitopertin phase II trials have started in the US and Australia (clinical trial.gov, ID NCT05308472). This drug is a glycine transporter inhibitor that aims to reduce blood PPIX concentrations and, consequently, phototoxicity. By inhibiting glycine transport into cells, it reduces the availability of glycine, a co‐substrate of ALAS2, the first and rate‐limiting enzyme of heme biosynthesis, which reduces the production of PPIX. Previously, bitopertin trials were conducted in patients with β‐thalassaemia and schizophrenia with a good safety profile.^66^, ^67^

#### 4.6.3 Bone marrow transplantation

Haematopoietic stem cell transplantation is an option for protoporphyria patients with severe liver complications.^68^ Successful HSCT can cure protoporphyria, preventing further liver damage and potential liver transplantation. However, it is important to acknowledge that HSCT is not always feasible and carries a significant risk of morbidity and mortality, particularly among adult patients. This presents a complex medical decision, as it involves weighing the risks and complications of HSCT against the estimated risk in each individual patient. Given the availability of current treatment options such as afamelanotide, we anticipate that very few patients and doctors will opt for HSCT in the absence of severe liver complications due to the risks involved.

### 4.7 Complications

The complications associated with the protoporphyrias are diverse and include protoporphyria‐related liver failure, gallstones, iron deficiency and vitamin D deficiency, with related reduced bone mineral density and fracture risk.

### 4.8 Liver disease and gallstones

**FIGURE 4:**
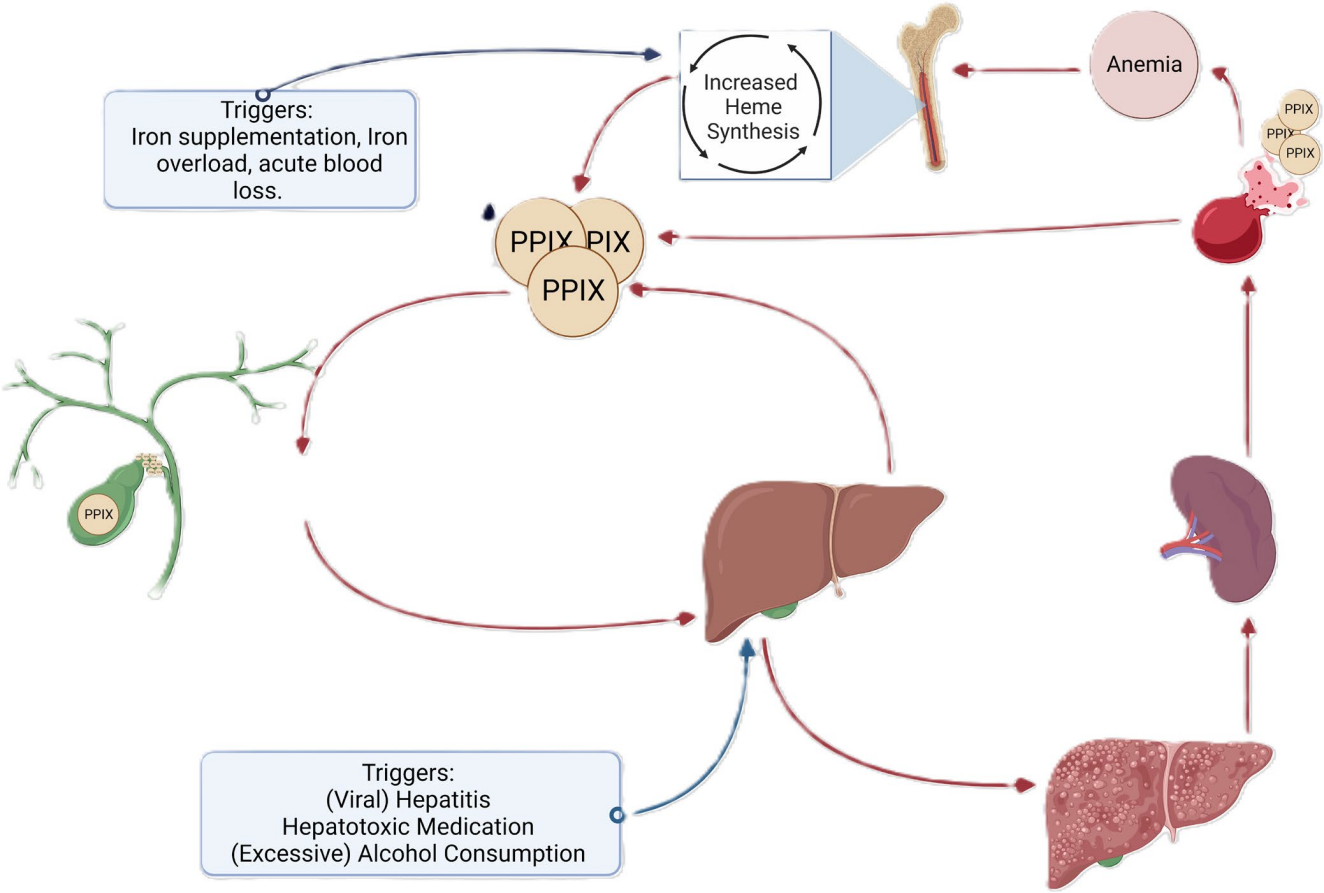
Pathophysiology of the progression of liver disease in protoporphyrias. PPIX levels accumulate not only in erythrocytes but also in bile ducts and the liver. This accumulation initiates a downward spiral, leading to cholestasis and decreased PPIX excretion, exacerbating hepatotoxicity. This liver damage ultimately results in fibrosis and acute cholestatic liver disease. A second downward spiral in severe cholestatic liver damage is the occurrence of splenomegaly and hypersplenism secondary to liver fibrosis, resulting in reduced erythrocyte survival. This, in turn, stimulates erythropoiesis, leading to a further increase in PPIX production. Both triggers that increase heme biosynthesis and/or induce hepatotoxicity can exacerbate these vicious cycles.

PPIX is a large, lipophilic molecule that is eliminated via bile excretion, it cannot be excreted in urine/water. In the intestine, PPIX is partially reabsorbed and is believed to enter the enterohepatic circulation. Deposition of excess PPIX in the hepatocytes, cholangiocytes and bile canaliculi can cause cholestatis in EPP patients. The latter reduces its biliary excretion of PPIX, which in turn may cause further accumulation, resulting in varying degrees of liver damage.^69^ With the progression of liver damage and impaired biliary PPIX excretion, PPIX increasingly accumulates in erythrocytes, plasma and the liver. This accumulation leads to a downward spiral, with exacerbating hepatotoxicity, leading to further liver damage and again lower PPIX excretion. Another exacerbating factor can be splenomegaly and hypersplenism, with shorter erythrocyte survival, which in turn stimulates erythropoiesis, leading to a further increase in PPIX production and again increased PPIX exposure in liver tissue (Figure 4).^70^, ^71^

The most dreaded, potentially life‐threatening complication of the protoporphyrias is cholestatic liver disease.^72^ As it may occur without early warning symptoms, most centres regularly monitor liver enzymes and perform liver ultrasound. The predictive value and the effect of monitoring on prognosis are uncertain.^38^ According to our expert opinion, we advise performing a baseline screening ultrasound as a reference point for all protoporphyria patients at the time of diagnosis. For individuals exhibiting no clinical symptoms and normal liver parameters, the evidence for the benefit of further routine ultrasounds is lacking.^73^ However, given the importance of early detection of liver disease in order to emphasize precautionary measures, ultrasounds can provide additional insights on liver status, including steatosis and fibrosis, which may not be detectable through laboratory parameters alone. Therefore, monitoring may be warranted for patients at risk. Such patients include those with high levels of PPIX,^22^, ^38^ those with XLEPP, those with the homozygous form of EPP1,^15^ those exposed to additional hepatotoxic factors or those with a family history of protoporphyria‐related liver disease.

**FIGURE 5:**
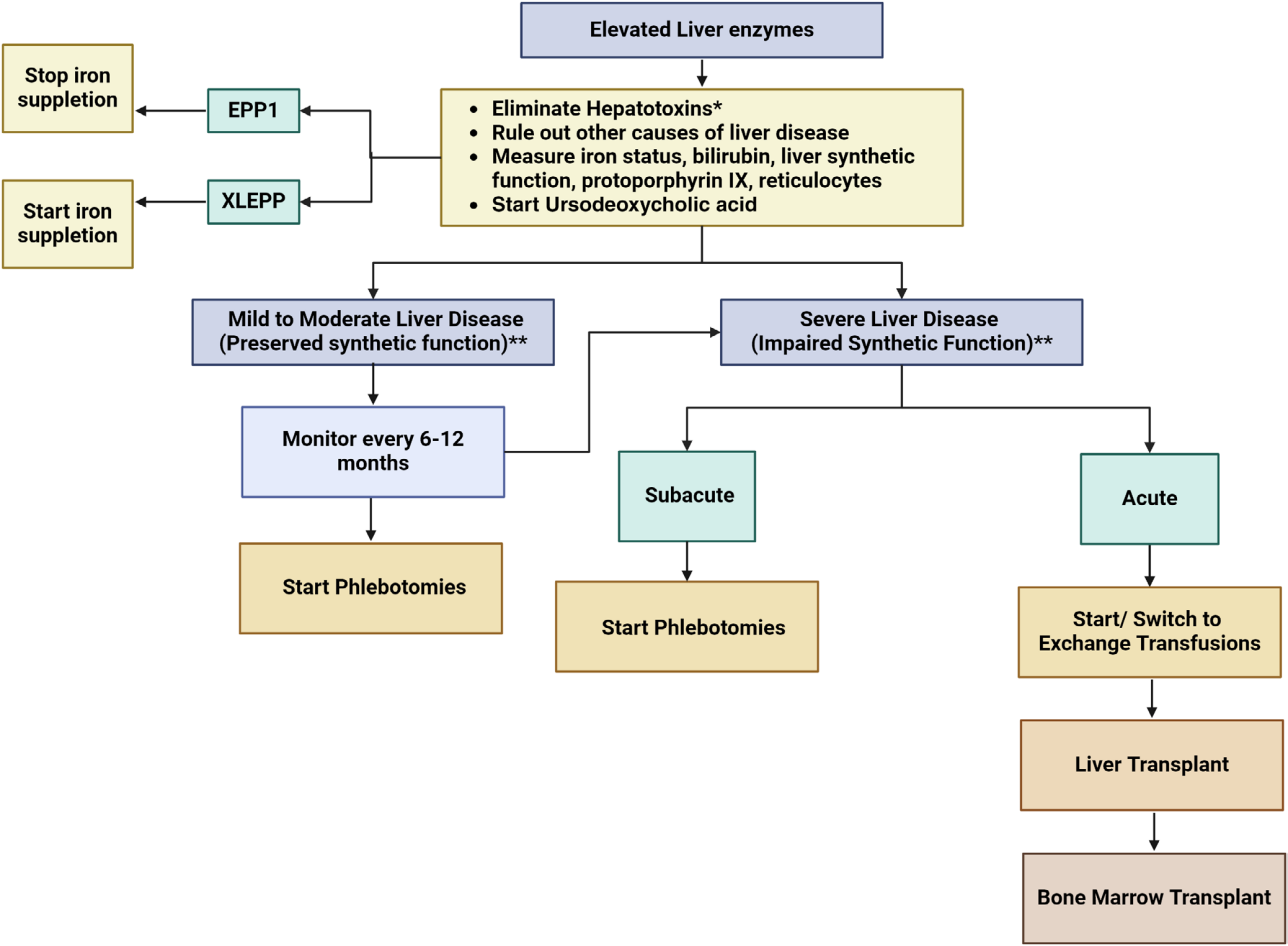
Management of protoporphyria‐related liver disease. Flowchart on the treatment of protoporphyria‐related liver disease was made based on the limited existing evidence and expert opinion. *Hepatotoxins include alcohol, oestrogens and iron supplementation in EPP1. **Liver synthetic function includes bilirubin, INR, albumin and ammoniac. Monitoring includes liver enzymes, bilirubin and an ultrasound of the liver. EPP1, erythropoietic protoporphyria.

The management of protoporphyria‐related liver disease depends on its severity and progression. Unfortunately, there is a paucity of evidence from interventions and long‐term follow‐up studies on this topic. Therefore, the following recommendations for protoporphyria‐related liver disease are based on case reports, case series,^73^ the authors' local clinical experience and guidelines for other liver diseases. For our suggested management of protoporphyria‐related liver disease, see also Figure 5.

#### 4.8.1 Mild to moderate liver disease

The prevalence of mild to moderate liver disease, defined by us as elevated liver enzymes with a preserved synthetic function, is approximately 6% in patients with protoporphyria.^74^ Significant liver fibrosis is present in approximately 10% of the population; the degree of liver fibrosis positively correlates with PPIX levels.^38^ The reason why some patients develop liver disease while others do not remains partly unknown. Although genetic causes have been suspected, to date, no confirmed relationship to specific mutations has been established.^75^, ^76^ Excessive alcohol consumption, hepatotoxic drugs or viral hepatitis have been suspected as potential precipitating factors^77^

In cases of mild to moderate liver disease, we recommend monitoring liver parameters, PPIX and liver ultrasound every 6–12 months. Liver elastography can be combined with the ultrasound to establish whether fibrosis is present.^73^ When other causes have been excluded, a liver biopsy is not considered necessary for confirming the diagnosis of protoporphyria‐related mild to moderate liver disease, as biopsies carry a considerable risk, like bleeding in up to 10%.^78^ Biopsies may, however, be indicated in selected cases to exclude additional causes of liver disease.^73^

As a preventive measure, all protoporphyria patients should be vaccinated against hepatitis A and B. As a first measure, any potential hepatotoxins, including alcohol and oestrogens, and, in patients with EPP1, iron supplementation should be eliminated. Contrary to EPP1, patients with XLEPP might benefit from iron supplementation, which can lower PPIX concentration and prevent or improve XLEPP‐related liver damage.^79^, ^80^, ^81^ In cases where hyperthyroidism is present, it should be treated.^82^

Due to the cholestatic nature of the protoporphyria‐related liver disease, treatment with ursodeoxycholic acid can be implemented, which is usually well tolerated.^83^, ^84^, ^85^, ^86^, ^87^ Additionally, the administration of cholestyramine could interrupt enterohepatic recirculation and reduce PPIX levels.^88^, ^89^, ^90^ The effect of intravenous vitamin E to reverse oxidative stress has been reported in a single case report.^91^ Observational studies have shown a dose‐dependent reduction of liver enzymes after long‐term treatment with afamelanotide.^74^, ^92^

To improve liver involvement in EPP1, low‐volume phlebotomies are currently tested in individual treatment attempts in some treatment centres and have been shown to effectively reduce iron stores, resulting in a reduction of PPIX accumulation.^93^, ^94^ Low volumes and strict monitoring are mandatory when starting phlebotomies in a protoporphyria patient, since as a result of phlebotomy, the erythroid heme synthesis may be enhanced, resulting in an increase in excess PPIX synthesis rates, depending on the iron stores.

#### 4.8.2 Severe liver disease

Severe liver disease affects 2%–5% of protoporphyia patients.^77^ Clinically, severe liver disease is defined by impaired synthetic liver function and is associated with jaundice, nausea, vomiting, abdominal pain in the right upper quadrant, increased photosensitivity and, in advanced cases, ascites and hepatic encephalopathy. Notably, severe hepatopathy has reportedly served as the initial sign leading to the diagnosis of protoporphyria in patients with a history of previously unexplained chronic photosensitivity.^49^ Although extremely rare, in patients with end‐stage liver disease, neurological symptoms similar to a progressive polyneuropathy with swallowing difficulties and respiratory depression have been described in protoporphyria.^35^

The following therapies can be considered to prevent further deterioration of severe acute progressive cholestatic liver disease. In many cases, a combination of these therapies is used to ‘bridge’ the patient to liver or curative bone marrow transplantation, with the main aim of reducing the body burden of excess PPIX. An overview of recommended treatment strategies, based on clinical expertise, is depicted in Figure 5.

Plasmapheresis,^93^, ^95^ erythrocyte exchange transfusions,^56^, ^70^, ^96^, ^97^, ^98^ both individually and in combination with haemodialysis,^99^ have been associated with a reduction in PPIX concentrations in case reports. Improvement, stabilization and prevention of recurrent liver disease with plasmapheresis before and after liver transplantation have been reported.^95^ One case report exploring various therapeutic approaches indicated that only erythrocyte exchange transfusions swiftly reduced PPIX levels in an acute setting.^98^

An alternative mode of action is suppressing erythropoiesis and subsequent PPIX production with blood transfusions,^97^ infusions of haematin,^100^, ^101^ or hydroxycarbamide.^98^ However, both haematin and blood transfusions supply an additional body iron burden, which may have adverse effects on the affected and inflamed liver in the long term. In one case of severe liver dysfunction, phlebotomies have been described as being effective in reversing the condition.^93^ Iron chelators have been used in another case report; however, hepatic toxicity should be taken into account.^98^

Liver transplantation is a rescue treatment for end‐stage cholestatic liver failure, but it does not cure protoporphyria. The five‐year survival rates of liver transplants are high.^102^ However, the incidence of recurrent cholestatic liver disease in transplant recipients is substantial, reaching 65%.^52^ Therefore, sequential bone marrow transplantation, which cures protoporphyria, should be considered^68^, ^103^ (Figure 5).

#### 4.8.3 Gallstones

PPIX changes the bile composition, leading to a risk of precipitation and gallstone formation. Therefore, gallstones are common in protoporphyrias, affecting up to 20%–25% of the patients.^35^, ^38^, ^104^, ^105^ Hence, a cholecystectomy may be warranted at a relatively young age.^104^, ^106^

### 4.9 Iron metabolism

The majority of patients with protoporphyria exhibit signs of an altered iron metabolism, with some patients having a mild to moderate anaemia with microcytosis and hypochromia and low serum ferritin and transferrin saturation.^107^, ^108^, ^109^ The exact pathophysiological mechanisms of the low iron state remain unexplained. Two studies on the iron metabolism in EPP1 showed that hepcidine, the master regulator of iron homeostasis, is not upregulated, thus enabling normal iron absorption in the intestine (Table 1).

However, XLEPP differs from EPP1 with respect to iron metabolism, as stated above. Here, the overly active ALAS2 enzymatic activity leads to an overproduction of PPIX in XLEPP, and, as FECH activity is normal, iron becomes the limiting factor for heme biosynthesis, resulting in an increase in ZnPP. Iron supplementation can reduce total and zinc‐bound PPIX.^37^, ^79^ Lower blood PPIX concentrations in turn reduce clinical symptoms^22^ and the risk for PPIX‐related liver damage.^22^, ^79^ Therefore, daily oral iron supplementation is recommended in XLEPP until a nadir in PPIX levels is achieved.^50^ Intravenous iron treatment can be considered in cases of intestinal iron malabsorption or oral iron intolerance. Based on the similarity of the disease mechanisms of EPP2 and XLEPP, the management of iron metabolism may be similar in the two conditions. However, due to the limited data available on EPP2, close monitoring of these patients is essential.

Contrary to XLEPP, EPP1 iron supplementation has been shown to increase PPIX, leading to increased photosensitivity and a higher risk for acute cholestatic liver disease.^37^, ^110^ This can be explained by the observation that in patients with EPP1, ALAS2 expression is upregulated both at the transcriptional and translational levels.^110^, ^111^ The translation of ALAS2 is regulated by the iron‐responsive element‐binding protein 2 (IRP2). Therefore, in an iron‐deficient state, the translation of the ALAS2 mRNA into protein is suppressed. Following iron substitution, the inhibition of ALAS2 by IRP2 is relieved, resulting in stimulation of the heme biosynthesis pathway with an increased production of PPIX (Figure 1).^111^ According to our clinical expertise, iron supplementation in EPP1 should only be prescribed restrictively, as we have observed significant increases in liver enzymes in several EPP1 cases a few weeks after regular doses of iron supplementation. In our clinical understanding, the only indication for iron supplementation is severe symptomatic iron deficiency anaemia, and we recommend that supplementation is best given at very low oral doses and with monitoring of liver parameters and PPIX values.

### 4.10 Vitamin D deficiency and osteoporosis

All protoporphyria patients are at risk for vitamin D deficiency as a result of their lifelong sun‐avoiding behaviour. In prior studies, the prevalence of vitamin D deficiency in protoporphyria increased, with up to half of the patients exhibiting Vitamin D3 levels <50 nmol/L.^29^, ^63^, ^112^, ^113^ In a Dutch cohort study, 36% of patients with EPP were diagnosed with osteopenia and 23% with osteoporosis, while the expected prevalence was 15% and 1%, respectively.^114^ Based on this high prevalence of osteopenia and osteoporosis, we consider one DEXA scan at adult age to evaluate bone mineral density, a conservative monitoring schedule.^114^ A recent study in the Dutch cohort shows that, despite receiving treatment with afamelanotide, which normalizes the time patients can spend outdoors, many patients remain deficient in vitamin D.^115^ Therefore, we recommend the prescription of vitamin D supplementation as part of supportive care for protoporphyria patients, even in cases of treatment with afamelanotide.^115^ The dosing can be based on current medical practice, followed by continued monitoring.

### 4.11 Contra‐indications for drug use

Hepatotoxic drugs should be avoided in protoporphyrias to prevent liver damage. Iron supplementation should be very cautiously given in EPP1. Yet, the warning on ‘porphyrinogenicity’ in the product summary of certain drugs is usually related to acute porphyrias and does therefore not apply to protoporphyrias.

### 4.12 Skin changes

Repeated phototoxic events may result in a permanently altered skin appearance on the areas of light‐exposed skin with a thickening or leathery appearance of the skin, including linear furrows or pseudorhagades of the lips, shallow comma‐shaped scarring, especially on the back of the nose, or a lichenification on the knuckles or the dorsa of the hands, as well as the loss of lunulae of fingernails.^35^, ^45^, ^116^, ^117^

### 4.13 Monitoring

Regular follow‐up of laboratory measurements should include, at a minimum, an annual assessment of blood count, iron status, liver enzymes, bilirubin, 25‐OH vitamin D and PPIX in erythrocytes. Monitoring and supplementation of 25‐OH vitamin D should be implemented to prevent a reduction in bone mineral density.^114^ Given the increased prevalence of osteopenia and osteoporosis in protoporphyria patients, at least one DEXA scan at adult age to evaluate bone mineral density should be considered.^114^

Since acute cholestatic liver disease is the most feared complication and can occur without warning, most centres will monitor liver enzymes and perform liver ultrasound. However, the predictive value and the effect of monitoring on prognosis are uncertain.^38^ It is advisable to regard a baseline screening ultrasound as a reference point for all protoporphyria patients. For individuals exhibiting no clinical symptoms and normal liver parameters, the evidence for the benefit of further routine ultrasounds is lacking.^73^ However, given the importance of early detection of liver disease, ultrasounds can provide additional insights on liver status, including steatosis and fibrosis, which may not be detectable through laboratory parameters alone. Therefore, monitoring may be warranted in patients at risk, for example, with especially high PPIX, additional hepatotoxic factors or a family history of liver disease.

## 5 CONCLUSION

The protoporphyrias are a group of three closely related ultra‐rare genetic diseases. Their management consists of the prevention of phototoxic reactions, if available, by treatment with the only approved drug, afamelanotide. Supporting the patients and their families in educating them about their social, school and work environments is an important part of care. In addition, long‐term consequences of light avoidance, such as vitamin D deficiency, bone health and mental health, need to be considered in the management of the patients. The most important complication of protoporphyrias is PPIX‐induced cholestatic liver disease. Due to its unpredictable and often sudden onset and the rarity of this complication, the evidence on how to optimally treat cholestatic liver disease is mostly based on case reports or case series, describing different approaches and treatment attempts. Based on the insights that iron supplementation might cause damage in EPP1 but improve outcomes in XLEPP, new approaches to prevent liver complications already in an early phase can be considered.

Rarely, HSCT, sometimes in combination with liver transplantation, has been used in patients with a high risk for liver failure; however, the approach is limited by the relatively high morbidity and mortality and low donor availability.

## FUNDING INFORMATION

Foundation of scientific research at the municipal hospital Zurich (Triemli).

## CONFLICT OF INTEREST STATEMENT

AEM: unrestricted research grant from Clinuvel Pharmaceuticals 2020–2021, support for a porphyria nurse by Alnylam Pharmaceuticals 2022–2023. LGK: no conflicts of interest. JBA: no conflicts of interest. EIM: consultant for Alnylam and Clinuvel. JGL: participates in contract studies for clinical trials with Ultragenyx Pharmaceutical, Clinuvel Pharmaceuticals Ltd and Alnylam® Pharmaceuticals (paid to her institution).
